# The Factors Associated with Attempted Smoking Cessation and Successful Four-Week Smoking Abstinence According to the Types of Disability in Seoul, Korea

**DOI:** 10.3390/ijerph18073548

**Published:** 2021-03-29

**Authors:** Han-Nu-Ri Kang, Kang-Sook Lee, JuYeon Koh, YuJin Park, HyunKyung Shin

**Affiliations:** 1 Seoul Tobacco Control Center, Seoul 06591, Korea; snfl@catholic.ac.kr (H.-N.-R.K.); hijoo@catholic.ac.kr (J.K.); rnyu@catholic.ac.kr (Y.P.); swhk@catholic.ac.kr (H.S.); 2 Department of Preventive Medicine, College of Medicine, The Catholic University of Korea, Seoul 06591, Korea

**Keywords:** type of disability, tobacco, smoking cessation counseling

## Abstract

This study investigated smoking behaviors by disability type among people with disabilities in Korea and identified factors associated with attempted smoking cessation and successful four-week smoking abstinence. Data were collected between 1 January 2018 and 31 December 2019. Predictors of attempted smoking cessation and successful four-week smoking abstinence were analyzed by disability type in 557 participants. Compared to people with mental health disorders, people with physical disabilities or brain lesions were more likely to attempt smoking cessation, and people with physical or internal disabilities were more likely to successfully abstain for four weeks. Common predictors of smoking cessation attempts and four-week abstinence were education level and CO level. Employment status predicted attempted cessation, while confidence in smoking cessation predicted four-week abstinence. To provide effective smoking cessation services for people with disabilities, disability type should be considered, and comprehensive and sustainable community-based programs need to be developed. Furthermore, a standardized survey of people with disabilities should be conducted to examine socioeconomic factors, including health status, employment, and education level, and to explore fundamental measures needed to address the problem of smoking among people with disabilities.

## 1. Introduction

The number of people with disabilities registered in Korea is growing, with 2,490,406 in 2015 and 2,618,918 in 2019, accounting for 5% of the entire Korean population [1]. Furthermore, the number of people with disabilities registered in Seoul increased from 391,753 in 2017 to 394,843 in 2019, accounting for approximately 4.1% of the city’s population (Seoul, 2020). With the growth of this population in Korea, needs have increased in various areas, including health and welfare, with related demands emerging in new forms. Particularly, less effort is made regarding disease prevention and health management for people with disabilities, compared to their non-disabled counterparts. Moreover, this population faces severe limitations related to their right to healthcare access, which negatively impacts their health [2].

A study using data from the 2013 Korea National Health and Nutrition Examination Survey (KNHANES) reported similar smoking rates between disabled and non-disabled populations, at 21.4% and 21.5%, respectively [3]. According to the 2017 National Survey on Persons with Disabilities, 58.7% of the population over 12 years of age “had never smoked a cigarette and currently do not smoke”, while 23% “used to smoke but not anymore”, indicating that 81.7% of the disabled population are current non-smokers. However, 15.7% claimed to “smoke every day” and 2.6% “smoke occasionally”, with a smoking rate of about 18.3% [4]. Furthermore, regarding smoking rates by disability type, most people with autism spectrum disorder or intellectual disorders were non-smokers, while many people with respiratory, cardiac, intestinal fistular, or urinary fistular dysfunction were former smokers. Among people with disabilities, the current smoking rate was highest in those with mental health disorders (39%) [4].

In the United States, most aspects of the health imbalance between people with and without disabilities are preventable, people with disabilities can and should continue healthy lives as people without disabilities, and they argued that reducing smoking is an important part of their healthy life [5]. However, according to a previous study, medical expenditures in the United States are increasing every year, and disability increases due to aging, and there is a high possibility that the gap in accessibility to medical care will worsen due to the gap between supply and demand [6]. Thus, as the average life expectancy increases and the registration rate of the disabled increases in the US as well as in Korea, there is a need for a customized smoking cessation service [7] to resolve the disparity in health status due to the existence of disabilities and continuous interest in health problems of the disabled. People with disabilities are likely to have reduced physical functioning and a high prevalence of early onset chronic disease [3], and related difficulties with mobility and communication can severely limit their healthcare access. Particularly, compared to the non-disabled population, people with disabilities experience more limited healthcare access, indicating they are seriously neglected by the healthcare system [8,9]. Therefore, society must pay attention to these inequalities experienced by people with disabilities, many of whom have limited healthcare utilization due to a lack of accessible facilities, financial problems, and communication difficulties [2].

Although smoking can be detrimental to the quality of life of people with disabilities, potentially exacerbating current disabilities and increasing the risk for a secondary disability or chronic disease, this is largely ignored by policies and institutions [10]. Further, the 2017 National Survey on Persons with Disabilities showed that only 2.4% of participants had received education for smoking prevention or cessation in the past year [4]. Despite reports that, compared with adults without disabilities, adults with disabilities are more likely to be smokers, they are less likely to be targeted for health interventions, education, or campaigns [11]. Moreover, smoking reduces the health-related quality of life of individuals and families and increases health and social expenditure [10].

Differences in data used in previous studies make it difficult to accurately compare smoking rates between people with and without disabilities; however, health-related issues for individuals with disabilities are relatively neglected in Korea, due to the prevailing social notion that “people with disabilities are unhealthy” [3]. Since cigarette prices increased in 2015, various smoking-related policies have been proposed and implemented, but most are tailored to individuals without disabilities, with a lack of studies and policies targeting those with disabilities.

Most previous studies on smoking among people with disabilities utilized data from either the KNHANES or National Survey on Persons with Disabilities. While systematic and direct health-promoting projects and relevant research are lacking, recent studies have shown that healthcare utilization and assistance or health-promoting behaviors by healthcare providers are correlated with successful smoking cessation in people with disabilities [12]. Thus, this study aimed to explore customized smoking cessation services for people with disabilities with limited healthcare access and identify predictors of attempted smoking cessation and successful four-week smoking abstinence by disability type.

## 2. Methods

### 2.1. Study Participants

Data were collected between 1 January 2018 and 31 December 2019 from the comprehensive smoking cessation information system. Participants included people with disabilities as defined by Chapter 3–32 of the Act on Welfare of Persons with Disabilities at the time of service registration. Data from 636 participants were obtained, and 577 with a disability clearly specified in the registry were included in the final analysis. This study was approved by the Institutional Review Board of the Catholic University of Korea (MC20ZASI0044).

### 2.2. Socio-Demographic Characteristics

Disability type, age, gender, education level, employment status, drinking habits, and exercise habits were analyzed as demographic characteristics. Disability type was classified according to the participant’s disability registered according to the Act on Welfare of Persons with Disabilities; for multiple disabilities, the most characteristic disability was chosen. “Brain lesions” refers to a complex disorders caused by brain damage, and “physical disabilities” refers to amputation disorder, physical dysfunction, and deformity. “Visual impairment” means a disability caused by damage to visual quality and visual fields, and “hearing impression” is a condition in which the ability to hear sounds is significantly impaired or not audible at all. “Internal disorders” include disorders of the kidneys, heart, liver, respiratory tract, intestines, and urinary tract. “Mental disorders” is developmental disorders, schizophrenia, bipolar affective disorder, and repetitive depressive disorder, etc. Education level was divided into uneducated/elementary school, middle school, high school, college, and “don’t know”/no response. Drinking habits were determined using a yes-or-no question asking about alcohol consumption in the past year. Exercise habits were determined using a yes-or-no question asking about moderate physical activity of 10 min or longer once a week.

### 2.3. Smoking-Related Characteristics

The smoking-related characteristics were measured: CO level, nicotine dependence, daily cigarette consumption, age at smoking initiation, total length of smoking, attempt smoking cessation, successful four-week smoking abstinence, number of counseling sessions, and “importance”, “confidence”, and “readiness” of motivation rulers were measured. Nicotine dependence was measured using the Fagerström test for nicotine dependence (FTND). Total scores range from 0 to 10, with higher scores indicating greater nicotine dependence, as follows: low dependence (0 ≤ FTND score ≤ 3), moderate dependence (4 ≤ FTND score ≤ 6), and high dependence (7 ≤ FTND score ≤ 10) [13]. Three motivation rulers were used to assess smoking cessation motivations. Importance was indexed by “How important is stopping smoking to you (0 = not important at all; 10 = most important goal in my life)?” Confidence was indexed by “How confident are you that you will quit smoking within the next month (0 = not at all; 10 = 100% confident)?” Readiness was indexed by “How ready are you to quit smoking within the next month (0 = not at all; 10 = 100% ready)?” [14].

### 2.4. Counseling Process

During the study period, smoking cessation education and campaigns and smoking prevention interventions were launched at a center for people with disabilities in Seoul to foster an environment for and promote active participation in smoking cessation services. Relevant facilities were recruited via telephone and wireless advertising, online advertising (blogs and Instagram), events for people with disabilities, and smoking cessation service promotion by attending personnel meetings. Additionally, services were provided in link to the mobile dental care provided by the Seoul Dental Hospital for the Disabled.

The smoking cessation campaign targeted all facility users, caregivers, and staff. Campaigns on vascular health and pulmonary capacity assessment, and events, such as smoking cessation roulette and smoking cessation trees, were launched to promote the service and foster an environment suitable to smoking cessation throughout facilities. Smoking prevention and cessation education programs were provided using audiovisual materials, such as pictures and videos specifically tailored to the audience. Particularly, education emphasized smoking hazards and etiquette and the importance of recognizing the problem of smoking and the need to quit. It also addressed other addictions, such as alcohol and gambling, and health-related content, including sexual health and obesity.

Prior to receiving smoking cessation counseling, participants signed informed consent forms detailing the study’s processing of personal information and disclosure of personal information to third parties, per the Personal Information Protection Act. After enrolling in the mobile smoking cessation service, at least nine individualized counseling sessions were provided; the number of sessions varied based on the facility or personal matters.

The counselor visited each facility and provided face-to-face counseling. If face-to-face counseling could not be provided, counseling was continued through phone calls or text messages. At each face-to-face counseling session, expiratory CO, vital capacity, and blood pressure were measured, and accumulated stress and vascular health were assessed once a month, to monitor participants’ physical health. Successful four-week smoking cessation was determined based on an expiratory CO level ≤ 6 ppm and negative urinary or salivary cotinine testing.

#### 2.4.1. Counseling for Visual Impairment

Prior to launching this project, an advisory meeting was held to establish a protocol for people with visual impairment, including preparing customized materials with braille and enlarged font. Further, counselors gained a better understanding of the disability, such as by learning about disability grading in advance, to build a rapport with people with visual impairment. Counselors also consistently practiced clear articulation and rehearsed a developed protocol to provide clear and detailed explanations.

#### 2.4.2. Counseling for Hearing Impairment

A professional sign language interpreter was present most sessions for people with hearing impairment. Visual materials containing pictures and text were used as much as possible. If an interpreter could not be present, counselors communicated through writing or the oral method. Counselors practiced basic sign language prior to sessions to help build a rapport with people with hearing impairments. Further, face-to-face counseling sessions were lengthened to ensure adequate counseling; when which face-to-face counseling could not be provided, counseling was continued through text messages or messenger applications (e.g., Kakaotalk (Kakao, Jeju-si, South Korea)).

#### 2.4.3. Counseling for Mental Health Disorders

People with mental health disorders showed poor concentration and understanding in many cases. To help these individuals focus on counseling, information about their disorder was collected in advance, based on a meeting with facility staff, and various activities were prepared. Particularly, data containing characters and simple images and videos were utilized. Information sheets on smoking, diet, oral health, and simple exercises were provided at each session to assist in health management. Moreover, to minimize resistance to smoking cessation counseling and promote active participation, activities such as health assessments and smoking cessation games were utilized during each session.

### 2.5. Statistical Analysis

Data were analyzed using SPSS 25.0 (IBM, Armonk, NY, USA). Differences in participant characteristics and smoking behaviors according to disability type were analyzed using chi-square tests and ANOVAs. Predictors of attempted smoking cessation and successful four-week cessation were identified using logistic regression.

## 3. Results

This section may be divided by subheadings. It should provide a concise and precise description of the experimental results, their interpretation, as well as the experimental conclusions that can be drawn.

### 3.1. General Characteristics

Most participants were male (*n* = 526; 94%), and brain lesions and physical disabilities were the most common. Mean participant age was 52.3 years. By disability type, mean age was the highest for physical disabilities (54.7 years), followed by brain lesions, internal disorders, hearing impairment, mental health disorders, and visual impairment. The most common education level was high school (33.2%), of which the majority were people with visual impairment or mental health disorders. Unlike other disability types, people with brain lesions tended to be well educated; 31% were college graduates or higher. In total, 51% of participants were employed; 18.9% did not answer this question. Among those with visual impairment, 64.3% were employed. In total, 53% of participants abstained from alcohol. Alcohol consumption was most common among those with visual impairment, followed by internal disorders, physical disabilities, mental health disorders, brain lesions, and hearing impairment. Among those with mental health disorders, 56.6% abstained from alcohol. Overall, 62.3% of participants engaged in at least 10 min of moderate activity once or more per week, especially those with brain lesions (69%) or hearing impairment (61.5%) (Table 1).

### 3.2. Smoking-Related Characteristics

#### 3.2.1. Smoking-Related Characteristics by Disability Type

Mean CO level was 8.66, and was highest among those with mental health disorders (12). In the brain lesion group, 59% had a CO level ≤ 6. Participants with mental health disorders had the highest percentage with a CO level between 11 and 20 (33.1%). Mean length of smoking was 33.5 years; total length of smoking was the highest in the brain lesion group (36.1 years). The overall rate of attempted cessation was 28.5%; this rate was the highest in the brain lesion group (41.8%). Overall, 13.3% of participants successfully abstained from smoking for four weeks; this rate was the highest in the brain lesion group (21.5%). Regarding counseling sessions, participants completed 3.22 on average; in the visual impairment group, 53.6% had at least three counseling sessions. Regarding motivation rulers, there were significant differences in confidence and readiness for smoking cessation. Mean confidence and readiness scores were 5.8 and 5.9, respectively, and these scores were the highest in the brain lesion group (6.4 and 6.6, respectively) (Table 2).

#### 3.2.2. Attempted Smoking Cessation and Successful Four-Week Smoking Abstinence by Smoking-Related Characteristics

Attempted smoking cessation and four-week smoking abstinence significantly differed according to CO level and number of counseling sessions. Overall, 159 participants attempted cessation. This group had a mean CO level of 5.7; 67.3% had a concentration ≤6. Furthermore, 74 participants successfully abstained from smoking for four weeks. This group had a mean CO level of 3.8; 82.4% had a CO concentration ≤6. Among those who attempted cessation and those who abstained from smoking for four weeks, participants attended, on average, 4.5 and 8 counseling sessions, respectively. In total, 49.1% of the attempted cessation group and 91.9% of the four-week abstinence group attended three or more counseling sessions (Table 3).

#### 3.2.3. Factors Associated with Attempted Smoking Cessation and Successful Four-Week Smoking Abstinence

Disability type, education level, employment status, and CO level were associated with attempted cessation. Compared to those with mental health disorders, participants with physical disabilities or brain lesions were 3.9 and 2.3 times more likely to attempt cessation, respectively. More educated participants were twice as likely to attempt cessation. Employed participants were 1.7 times more likely to attempt cessation; those with low CO levels were 3.3 more likely. The odds for a successful four-week smoking abstinence were 5.3 times higher among those with physical disabilities, compared to those with mental health disorders, and 4.2 times higher among those with internal disorders. The odds of successfully abstaining from smoking for four weeks were 3.2 times higher among more educated participants and 5.2 times higher among those with low CO levels. Regarding the motivation ruler, those with higher confidence in their smoking cessation ability were 1.18 times more likely to successfully abstain from smoking for four weeks (Table 4).

## 4. Discussion

In this study, people with disabilities were provided customized smoking cessation services, to examine smoking-related behaviors by disability type and identify predictors of attempted cessation and successful four-week smoking abstinence. Overall, 28.5% of participants attempted smoking cessation, and 13.3% successfully abstained from smoking for four weeks. Particularly, people with physical disabilities or brain lesions were more likely to attempt cessation than those with mental health disorders, while people with physical disabilities or internal disorders were more likely to successfully abstain from smoking for four weeks. Brain lesions had the highest level of confidence and readiness in motivation runners compared to other types of disabilities. Direct comparison is difficult as there is no study in the same target group as this study, but the results are similar to previous studies that motivation rulers influenced long-term cessation success in a study of adult males [14,15]. Internal and physical disabilities were among groups with higher number of consultations or lower CO levels than other disability types. The result is similar to the study that the higher the number of counseling to quit smoking and the lower the CO level, the higher the cessation success rate [16]. This differed from the 2019 Statistical Report for Persons with Disabilities, in which rates of attempted smoking cessation were higher among those with a facial disability (38.7%) or respiratory disorder (35.8%), compared with the other 13 disability types among 2.6 million registered individuals [17].

In the present study, those who were high school graduates or higher were more likely to attempt smoking cessation and successfully abstain from smoking for four weeks, compared to those with less education. This was similar to previous findings that the odds of successful smoking cessation were higher with increasing education level [10]. Additionally, employed participants were more likely to attempt smoking cessation, compared with those who were unemployed or did not respond to this question. Considering that employment provides opportunities for people with disabilities to not only maintain their physical and mental health but also find a social support network to help them quit smoking, this was in line with previous findings that regular physical activity helps people with disabilities maintain independent functioning, promotes positive recovery, and prevents secondary health problems related to one’s disability [18].

This study found that the odds of attempting smoking cessation and successfully maintaining short-term abstinence increased with decreasing CO concentration. Participants with lower CO levels were more likely to attempt cessation and successfully abstain from smoking for four weeks, compared to those with a concentration of 7 ppm or higher. Unlike urinary and blood testing, measuring expiratory CO is noninvasive and provides immediate results [19]; thus, it is useful in environments where cotinine testing is difficult. By checking expiratory CO concentration immediately during face-to-face counseling, participants can acknowledge that the results are caused by smoking, which can be utilized as a means to further motivate them to quit smoking by linking it to their health.

People with disabilities who engaged in more smoking cessation counseling sessions were more likely to attempt smoking cessation and successfully abstain for four weeks. While most smokers attempt smoking cessation on their own, many fail, and assistance is crucial. The odds for success increased when participants attended a higher number of counseling sessions [20], and increased rates of successful cessation were associated with appropriately trained healthcare providers, longer counseling duration, and multidisciplinary assistance provided during counseling [21]. Further, concerns regarding additional loss of functioning may influence people with physical disabilities or chronic illnesses to be more strongly motivated to quit smoking [22]. People with disabilities are highly likely to consume various tobacco products, which increases the risk of adverse health effects [23,24]. Particularly, people with disabilities are disadvantaged by a shortage of accessible smoking cessation programs in relation to their smoking rate [25], highlighting the importance of providing individualized smoking cessation services.

While the results of the present study pertaining to nicotine dependence were not significant, a previous study reported that people with disabilities with high nicotine dependence are at risk of consuming multiple cigarettes and may face challenges when quitting smoking [23], indicating the need for further research. Specifically, people with mental health disorders showed an extremely high smoking rate and nicotine dependence [26]; thus, providing customized, varied, and aggressive smoking cessation services according to disability type could markedly reduce the smoking rate among people with disabilities.

This study observed that alcohol non-users were less likely to attempt smoking cessation or successfully abstain from smoking for four weeks compared to alcohol users; however, these results were not significant. This contradicts previous findings that alcohol consumption has a positive effect on smoking in people with disabilities [3,10,27]. Moreover, in light of prior findings that health-related concerns and behaviors increase with advanced age, and thus smoking cessation attempts consequently increase [10], it is necessary to further examine the impact of age and alcohol consumption on the smoking rate for people with disabilities.

Motivation rulers were not significantly associated with continued smoking abstinence, and only the confidence ruler was associated with successful four-week smoking abstinence. This was slightly different from the findings of a previous study with female Korean undergraduates, in which motivation rulers predicted most instances of successful cessation [28]. These results suggested that improving counselors’ skills and implementing effective activities to promote confidence in one’s ability to quit smoking could help people with disabilities attempt and maintain smoking abstinence over both the short and long term.

Despite growing interest in smoking cessation services for people with disabilities, numerous obstacles hinder this population’s access to such services [23]. Based on the findings that barriers and environmental factors should be considered [10], it should be acknowledged that disability-specific smoking cessation services are essential health and welfare services. Thus, such services should not only focus on providing smoking cessation counseling but also tailor it to the needs related to specific disabilities [29], to ensure individuals with disabilities have the same healthcare access as non-disabled people, thereby improving their quality of life.

Although participants were classified by disability type to provide customized smoking cessation services, this study has a few limitations and requires caution when interpreting the results. First, this study was conducted on people with disabilities living in Seoul, the findings cannot be generalized to other populations. Second, we could not examine participants’ socioeconomic factors. Previous studies showed that socioeconomic status may make a greater contribution to increasing the smoking rate among people with disabilities [30]. Given that education level, income level, employment status, and employment type are factors that may affect health behaviors and health status, not only helping people improve management of their smoking rate but also improving their socioeconomic status may be an important intervention to lower the smoking rate among people with disabilities [31].

In the smoking cessation services for people with disabilities, different approaches were taken based on disability type, such as strengthening motivation to quit smoking by emphasizing its direct connection to health and establishing a protocol in advance through advisory meetings. However, the smoker population in the recruited facilities showed little interest in smoking cessation, and their caregivers and staff had no relevant knowledge. Even those enrolled in smoking cessation services faced a number of difficulties in maintaining smoking abstinence, such as frequent deterioration in vigor due to medications, habitual smoking due to insomnia, and smoking to relieve pain. In the future, studies should include more detailed analysis and establish protocols for each disability type to promote six-month abstinence from smoking among people with disabilities.

Despite these limitations, this study was the first to utilize data obtained by providing visiting smoking cessation services for people with disabilities. This study is significant in that it analyzed smoking behaviors and related predictors disability type, as well as predictors of attempted and successful smoking cessation. We expect that our results could provide valuable foundational data for developing and improving effective smoking cessation services for people with disabilities.

## 5. Conclusions

While people with disabilities have high smoking rates, they often do not have access to proper healthcare services, and relevant research is lacking. The purpose of this study was to examine smoking behaviors in people with disabilities in Korea by type of disability and identify the factors associated with attempted smoking cessation and successful four-week smoking abstinence. The results showed that the type of disability, education level, and CO level were predictors of both attempted smoking cessation and successful four-week smoking abstinence, with employment status associated with attempted smoking cessation and confidence in quitting smoking associated with successful four-week smoking abstinence.

To provide effective smoking cessation services for people with disabilities, their disability type and environment must be taken into consideration. Moreover, comprehensive and sustainable programs need to be developed, such as increasing smoking cessation awareness in those who provide support to people with disabilities, such as caregivers and facility staff, by emphasizing the harm caused by and need to quit smoking among people with disabilities, and establishing a network among facilities in the community to improve this population’s access to smoking cessation services. Additionally, it is crucial that policy measures to monitor the status of people with disabilities are implemented. For example, it is important to conduct a standardized survey of people with disabilities to examine their overall status, including health status, employment status, and education level, and expend efforts to boost their standard of living to that of the non-disabled population in order to tackle fundamental problems related to smoking in people with disabilities.

## Figures and Tables

**Table 1:** General characteristics according to disability types.

Variable		Disability Types (*n* = 557)							
		Brain Lesions *N* = 200(%)	Physical Disabilities *N* = 137(%)	Visual Impairment *N* = 28(%)	Hearing Impairment *N* = 13(%)	Internal Disorders *N* = 34(%)	Mental Disorders *N* = 145(%)	557(%) or M ± SD	*p*
**Gender**									
Female		5 (2.5)	8 (5.8)	2 (7.1)	1 (7.7)	2 (5.9)	13 (9)	31 (5.6)	0.218
Male		195 (97.5)	129 (94.2)	26 (92.9)	12 (92.3)	32 (94.1)	132 (91)	526 (94.4)	
**Age**	**M ± SD**	54.36 ± 13.58	54.72 ± 13.21	46.00 ± 15.40	49.23 ± 14.85	54.24 ± 12.31	48.01 ± 15.36	52.25 ± 14.32	<0.001
≤20		1 (0.5)	1 (0.7)	0 (0.0)	0 (0.0)	12 (8.3)	0 (0.0)	14 (2.5)	<0.001
21–30		8 (4.0)	7 (5.1)	4 (14.3)	1 (7.7)	13 (9.0)	2 (5.9)	35 (6.3)	
31–40		27 (13.5)	12 (8.8)	6 (21.4)	3 (23.1)	12 (8.3)	3 (8.8)	63 (11.3)	
41–50		36 (18.0)	28 (20.4)	9 (32.1)	3 (23.1)	34 (23.4)	2 (5.9)	112 (20.1)	
51–60		56 (28.0)	44 (32.1)	5 (17.9)	3 (23.1)	46 (31.7)	19 (55.9)	173 (31.1)	
61–70		50 (25.0)	30 (21.9)	1 (3.6)	2 (15.4)	19 (13.1)	5 (14.7)	107 (19.2)	
≥71		22 (11.0)	15 (10.9)	3 (10.7)	1 (7.7)	9 (6.2)	3 (8.8)	53 (9.5)	
**Education level**									
Uneducated or elementary education		13(6.5)	16 (11.7)	0 (0.0)	1 (7.7)	3 (8.8)	14 (9.7)	47 (8.4)	0.015
Middle		21 (10.5)	18 (13.1)	3 (10.7)	3 (23.1)	1 (2.9)	14 (9.7)	60 (10.8)	
High		66 (33.0)	42 (30.7)	13 (46.4)	3 (23.1)	10 (29.4)	51 (35.2)	185 (33.2)	
College		63 (31.5)	29 (21.2)	6 (21.4)	0 (0.0)	7 (20.6)	22 (15.2)	127 (22.8)	
No response		37(18.5)	32 (23.4)	6 (21.4)	6 (46.2)	13 (38.2)	44 (30.3)	138 (24.8)	
**Employment status**									
Yes		75 (37.5)	37 (27.0)	18 (64.3)	2 (15.4)	9 (26.5)	32 (22.1)	173 (31.1)	<0.001
No		93 (46.5)	75 (54.7)	8 (28.6)	4 (30.8)	19 (55.9)	80 (55.2)	279 (50.1)	
No response		32(16.0)	25 (18.2)	2 (7.1)	7 (53.8)	6 (17.6)	33 (22.8)	105 (18.9)	
**Drinking**									
No		118 (59.0)	64 (46.7)	10 (35.7)	8 (61.5)	13 (38.2)	82 (56.6)	295 (53.0)	0.028
Yes		82 (41.0)	73 (53.3)	18 (64.3)	5 (38.5)	21 (61.8)	63 (43.4)	262 (47.0)	
**Exercise**									
No		62 (31.0)	56 (40.9)	11 (39.3)	5 (38.5)	17 (50.0)	59 (40.7)	210 (37.7)	0.207
Yes		138 (69.0)	81 (59.1)	17 (60.7)	8 (61.5)	17 (50.0)	86 (59.3)	347 (62.3)	

**Table 2:** Smoking-related characteristics according to disability types.

Variable		Disability Types (*n* = 557)							
		Brain Lesions *N* = 200(%)	Physical Disabilities *N* = 137(%)	Visual Impairment *N* = 28(%)	Hearing Impairment *N* = 13(%)	Internal Disorders *N* = 34(%)	Mental Disorders *N* = 145(%)	Total *N* = 557(%)	*p*
**CO level (ppm)**	M ± SD	6.79 ± 6.50	7.91 ± 6.02	9.54 ± 7.06	7.31 ± 4.82	7.94 ± 6.31	12.08 ± 8.27	8.66 ± 7.18	<0.001
0~6		118 (59.0)	57 (41.6)	12 (42.9)	6 (46.2)	16 (47.1)	39 (26.9)	248 (44.5)	<0.001
7~10		41 (20.5)	42 (30.7)	6 (21.4)	3 (23.1)	7 (20.6)	36 (24.8)	135 (24.2)	
11~20		31 (15.5)	32 (23.4)	7 (25.0)	4 (30.8)	9 (26.5)	48 (33.1)	131 (23.5)	
≥21		10 (5.0)	6 (4.4)	3 (10.7)	0 (0.0)	2 (5.9)	22 (15.2)	43 (7.7)	
**Nicotine dependence**	M ± SD	3.38 ± 2.57	3.61 ± 2.60	3.14 ± 2.17	3.77 ± 2.71	4.21 ± 2.48	3.81 ± 2.34	3.60 ± 2.50	0.208
Low (0~3)		99 (49.5)	61 (44.5)	17 (60.7)	6 (46.2)	12 (35.3)	60 (41.4)	255 (45.8)	0.627
Mild (4~6)		77 (38.5)	61 (44.5)	8 (28.6)	6 (46.2)	17 (50.0)	62 (42.8)	231 (41.5)	
High (≥7)		24 (12.0)	15 (10.9)	3 (10.7)	1 (7.7)	5 (14.7)	23 (15.9)	71 (12.7)	
**Daily cigarette consumption**	M ± SD	14.57 ± 9.97	14.23 ± 9.31	13.61 ± 8.04	18.23 ± 14.45	15.15 ± 7.77	15.54 ± 9.92	14.81 ± 9.70	0.294
0–4		30 (15.0)	15 (10.9)	4 (14.3)	0 (0.0)	2 (5.9)	18 (12.4)	69 (12.4)	0.533
5–9		29 (14.5)	23 (16.8)	3 (10.7)	3 (23.1)	4 (11.8)	17 (11.7)	79 (14.2)	
10–14		44 (22.0)	28 (20.4)	8 (28.6)	3 (23.1)	9 (26.5)	33 (22.8)	125 (22.4)	
15–19		9 (4.5)	19 (13.9)	3 (10.7)	1 (7.7)	4 (11.8)	10 (6.9)	46 (8.3)	
≥20		88 (44.0)	52 (38.0)	10 (35.7)	6 (46.2)	15 (44.1)	67 (46.2)	238 (42.7)	
**Age of first smoking (year)**	M ± SD	19.33 ± 4.96	20.74 ± 7.34	19.68 ± 3.52	20.62 ± 5.01	20.62 ± 8.78	20.19 ± 5.81	20.03 ± 6.07	0.338
0–12		6 (3.0)	6 (4.4)	0 (0.0)	0 (0.0)	0 (0.0)	7 (4.8)	19 (3.4)	0.489
13–15		31 (15.5)	12 (8.8)	2 (7.1)	1 (7.7)	7 (20.6)	11 (7.6)	64 (11.5)	
16~18		55 (27.5)	37 (27.0)	7 (25.0)	4 (30.8)	9 (26.5)	37 (25.5)	149 (26.8)	
≥19		108 (54.0)	82 (59.9)	19 (67.9)	8 (61.5)	18 (52.9)	90 (62.1)	325 (58.3)	
**Smoking period (year)**	M ± SD	36.18 ± 13.86	35.27 ± 14.32	27.86 ± 15.77	30.08 ± 13.89	34.50 ± 14.80	29.50 ± 15.37	33.55 ± 14.79	<0.001
**Smoking quit attempt**									
No		117 (58.5)	97 (70.8)	23 (82.1)	12 (92.3)	26 (76.5)	123 (84.8)	398 (71.5)	<0.001
Yes		83 (41.5)	40 (29.2)	5 (17.9)	1 (7.7)	8 (23.5)	22 (15.2)	159 (28.5)	
**Successful 4 week smoking**									
No		157 (78.5)	124 (90.5)	24 (85.7)	12 (92.3)	28 (82.4)	138 (95.2)	483 (86.7)	<0.001
Yes		43 (21.5)	13 (9.5)	4 (14.3)	1 (7.7)	6 (17.6)	7 (4.8)	74 (13.3)	
**Frequency of counseling**	M ± SD	3.53 ± 4.03	2.80 ± 2.87	5.50 ± 4.74	1.38 ± 0.65	3.03 ± 3.36	2.95 ± 2.81	3.22 ± 3.48	0.001
1		84 (42.2)	53 (38.7)	7 (25.0)	9 (69.2)	15 (44.1)	54 (37.2)	222 (39.9)	0.081
2		44 (22.1)	42 (30.7)	6 (21.4)	3 (23.1)	8 (23.5)	41 (28.3)	144 (25.9)	
≥3		71 (35.7)	42 (30.7)	15 (53.6)	1 (7.7)	11 (32.4)	50 (34.5)	190 (34.2)	
**Motivation rulers**									
Importance	M ± SD	8.09 ± 2.31	7.74 ± 2.49	7.68 ± 2.34	7.46 ± 3.60	8.06 ± 2.39	7.32 ± 2.52	7.77 ± 2.46	0.114
Confidence	M ± SD	6.40 ± 2.86	5.82 ± 3.01	5.75 ± 3.07	6.38 ± 3.18	5.38 ± 2.88	5.32 ± 2.68	5.88 ± 2.89	0.019
Readiness	M ± SD	6.61 ± 2.96	6.09 ± 3.12	5.64 ± 3.13	5.85 ± 3.31	5.18 ± 2.81	5.32 ± 2.83	5.99 ± 3.01	0.002

ppm: parts per million, which stands for carbon monoxide concentration.

**Table 3:** Attempted smoking cessation and successful four-week smoking abstinence by smoking-related characteristics.

Variable		Total *N* = 557(%)	Attempted Smoking Cessation			Successful 4-Week Smoking Abstinence		
			No *N* = 398(%)	Yes *N* = 159(%)	*p*	No *N* = 483(%)	Yes *N* = 74(%)	*p*
**CO level (ppm)**	M ± SD	8.66 ± 7.18	9.83 ± 7.15	5.75 ± 6.41	<0.001	9.40 ± 7.08	3.84 ± 5.88	<0.001
0~6		248 (44.5)	141 (35.4)	107 (67.3)	<0.001	187 (38.7)	61 (82.4)	<0.001
7~10		135 (24.2)	113 (28.4)	22 (13.8)		127 (26.3)	8 (10.8)	
11~20		131 (23.5)	107 (26.9)	24 (15.1)		128 (26.5)	3 (4.1)	
≥21		43 (7.7)	37 (9.3)	6 (3.8)		41 (8.5)	2 (2.7)	
**Nicotine dependence**	M ± SD	3.60 ± 2.50	3.61 ± 2.43	3.57 ± 2.68	0.856	3.64 ± 2.44	3.34 ± 2.86	0.395
Low (0~3)		255 (45.8)	181 (45.5)	74 (46.5)	0.313	217 (44.9)	38 (51.4)	0.216
Mild (4~6)		231 (41.5)	171 (43.0)	60 (37.7)		207 (42.9)	24 (32.4)	
High (≥7)		71 (12.7)	46 (11.6)	25 (15.7)		59 (12.2)	12 (16.2)	
**Cigarettes per day**	M ± SD	14.81 ± 9.70	14.51 ± 9.13	15.55 ± 10.98	0.291	14.82 ± 9.38	14.76 ± 11.66	0.966
0–4		69 (12.4)	43 (10.8)	26 (16.4)	0.054	54 (11.2)	15 (20.3)	0.106
5–9		79 (14.2)	60 (15.1)	19 (11.9)		68 (14.1)	11 (14.9)	
10–14		125 (22.4)	98 (24.6)	27 (17.0)		115 (23.8)	10 (13.5)	
15–19		46 (8.3)	36 (9.0)	10 (6.3)		41 (8.5)	5 (6.8)	
≥20		238 (42.7)	161 (40.5)	77 (48.4)		205 (42.4)	33 (44.6)	
**Age of first smoking (year)**	M ± SD	20.03 ± 6.07	20.02 ± 6.03	20.05 ± 6.18	0.954	20.14 ± 6.26	19.27 ± 4.60	0.250
0–12		19 (3.4)	13 (3.3)	6 (3.8)	0.434	17 (3.5)	2 (2.7)	0.207
13–15		64 (11.5)	47 (11.8)	17 (10.7)		55 (11.4)	9 (12.2)	
16~18		149 (26.8)	99 (24.9)	50 (31.4)		122 (25.3)	27 (36.5)	
≥19		325 (58.3)	239 (60.1)	86 (54.1)		289 (59.8)	36 (48.6)	
**Frequency of counseling**	M ± SD	3.22 ± 3.48	2.71 ± 2.90	4.50 ± 4.36	<0.001	2.48 ± 2.67	8.07 ± 4.20	<0.001
1		223 (40.0)	163 (41.1)	59 (37.1)	<0.001	222 (46.1)	0 (0.0)	<0.001
2		144 (25.9)	122 (30.7)	22 (13.8)		138 (28.6)	6 (8.1)	
≥3		190 (34.1)	112 (28.2)	78 (49.1)		122 (25.3)	68 (91.9)	

ppm: parts per million, which stands for carbon monoxide concentration.

**Table 4:** The factors associated with attempted smoking cessation and successful four-week smoking abstinence.

Variable	Attempted Smoking Cessation	Successful 4-Week Smoking Abstinence
	Odds Ratio (95% CI)	Odds Ratio (95% CI)
**Disability types**		
Mental health disorders	1	1
Physical disabilities	3.97 (2.32–6.76) ***	5.40 (2.35–12.39) ***
Brain lesions	2.30 (1.29–4.14) **	2.07 (0.80–5.35)
Visual impairment	1.22 (0.42–3.54)	3.29 (0.89–12.09)
Hearing impairment	0.47 (0.06–3.77)	1.64 (0.19–14.49)
Internal disorders	1.72 (0.69–4.29)	4.22 (1.32–13.53) *
**Drinking**		
Yes	1	1
No	0.98 (0.66–1.47)	0.70 (0.40–1.22)
**Education level**		
≤Middle school	1	1
≥High school	2.01 (1.37–2.97) ***	3.27 (1.83–5.85) ***
**Employment status**		
No	1	1
Yes	1.79 (1.21–2.63) **	1.62 (0.98–2.68)
**CO Level**		
≥7	1	1
0–6	3.32 (2.18–5.06) ***	5.20 (2.68–10.09) ***
**Total smoking length**		
≥20 year	1	1
≤20 year	0.95 (0.55–1.62)	0.92 (0.45–1.91)
**Motivation rulers**		
Importance	1.05 (0.95–1.15)	1.04 (0.90–1.19)
Confidence	1.06 (0.96–1.16)	1.18 (1.02–1.37) *
Readiness	1.05 (0.95–1.15)	1.08 (0.93–1.24)

* *p* < 0.05, ** *p* < 0.01, *** *p* < 0.001.

## Data Availability

The data supporting the findings of this study are available only to researchers who participated in 17 Tobacco Control Centers in Korea. The data are not publicly available due to privacy.
